# Synthesis and evaluation of a prodrug of 5-aminosalicylic acid for the treatment of ulcerative colitis

**DOI:** 10.22038/IJBMS.2019.13991

**Published:** 2019-12

**Authors:** Yan Yan, Fengling Ren, Pengchong Wang, Ying Sun, Jianfeng Xing

**Affiliations:** 1 School of Pharmacy, Xi’an Jiaotong University, Xi’an, Shaanxi, China; 2 School of Public Health, Xi’an Jiaotong University, Xi’an, Shaanxi, China

**Keywords:** Anti-inflammation, Colon targeting, Prodrug, Ulcerative colitis, 5-Aminosalicylic acid

## Abstract

**Objective(s):**

This study is aimed to design and synthesize a prodrug of 5-aminosalicylic acid and evaluate its ameliorative effect on experimental ulcerative colitis (UC).

**Materials and Methods:**

5-Aminosalicylic acid-alanine (5-ASA-ALA) was synthesized and characterized. Its stability study was conducted in rat plasma and in the gastrointestinal tract environment, its transport characteristic was assessed using the Caco-2 cells. Its colon-targeting property was evaluated by the pharmacokinetic study, and incubation studies. A series of indicators were used to investigate its therapeutic effect on experimental colitis, including the survival rate and body weight of mice, the disease activity index (DAI), the colonic damage score and colon index, the myeloperoxidase (MPO) activity and the levels of malondialdehyde (MDA), total superoxide dismutase (SOD), glutathione (GSH) and glutathione peroxidase (GSH-Px) in colonic tissues.

**Results:**

5-ASA-ALA was barely absorbed in the Caco-2 monolayer or into the rat blood. It was remarkably stable when incubated in the upper gastrointestinal tract, while gradually hydrolyzed in the colon of rats. When orally administered to mice, 5-ASA-ALA had significantly greater therapeutic effect on colitis than the positive control.

**Conclusion:**

5-ASA-ALA is demonstrated to be a promising oral colon-targeting prodrug of 5-ASA and has potential application in UC treatment.

## Introduction

5-Aminosalicylic acid (5-ASA) is a kind of non-steroid anti-inflammatory drug, which is widely used for mild to moderate inflammatory bowel disease therapy. 5-ASA is also regarded as an antioxidant for an amino derivative of salicylic acid, it is used for trapping free radicals that possibly damaged the metabolic by-products. Particularly, 5-ASA is effectively and commonly used in the treatment of ulcerative colitis (UC) (1, 2). UC is a chronic inflammatory disorder of the intestinal tract, the pathogenesis of which is complicated and undefined, the most important symptom is chronic inflammation of the gastrointestinal tract with periods of exacerbation followed by intervals of remission, and its commonly used medications are 5-ASA, steroids, and immunosuppressant (3, 4). Over the last few decades, therapy for UC has focused on 5-ASA. It has been shown that repeated and high dosage of 5-ASA is essential for helping with symptoms, maintaining remission and preventing relapse of UC (5).

 However, 5-ASA is almost completely and rapidly absorbed from the proximal small intestine after oral administration, thus greatly reducing the drug delivered specifically to the colon (6). Besides, the clinical application of free 5-ASA is limited by its complete and rapid absorption from the upper digestive tract, which depended on the local pH value and concentration (7). Therefore, it is of great importance to directly deliver 5-ASA to the colon and to decrease its systemic absorption and improve the possibility for therapeutic success. Prodrug is an effective method for colon-targeting as reflected in the clinical success of azo prodrugs such as olsalazine (8). So far efforts have been made to prepare oral formulations of 5-ASA, therefore, prodrugs of 5-ASA have been designed and prepared with various chemical bonds such as azo bonds, glycosides bonds, ester bonds and amide bonds. The synthesized prodrugs would be specifically activated by the colonic microflora in the gastrointestinal transit (9, 10). Especially, prodrugs of 5-ASA modified by hydrophilic small molecules through amide bonds could be hydrolyzed by the colonic microflora, thus possessing colon-targeting property (11). As a desirable oral colon-specific drug delivery system, an amide prodrug of 5-ASA is supposed to be biochemically stable, has high efficacy in minimizing the side effects of 5-ASA caused by systemic absorption while low absorption in upper digestive tract (12).

**Scheme 1:**
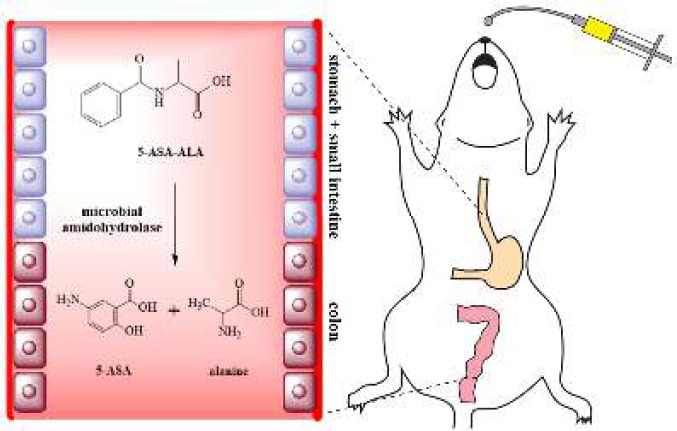
A prodrug of 5-aminosalicylic (5-ASA-ALA) acid was designed and synthesized in this study. 5-aminosalicylic (5-ASA-ALA) was proved to have desirable colon-targeting property and therapeutic effect on experimental colitis when orally administrated to mice

In this study, a colon-targeting prodrug of 5-ASA was prepared by combined with alanine through an amidation reaction with simple synthetic route, and characterized by ATR-FTIR and ^1^H NMR. As a natural component, alanine is nontoxic and free from any side effects, it is relatively cheap and easily available. The colon-targeting property of the synthesized prodrug, 5-aminosalicylic acid-alanine (5-ASA-ALA), was evaluated by a series of studies, including the pharmacokinetic study, Caco-2 transport study, the *in vitro* and* in vivo* incubation study. Besides, the ameliorative effect of 5-ASA-ALA was systematically evaluated by several biological indicators and compared with a positive control (a mixture of 5-ASA and alanine) in mice with experimental colitis (Scheme 1).

## Materials and Methods

***Materials***

5-Nitrosalicylic acid (5-NSA), alanine methyl ester, L-alanine, palladium on activated charcoal (Pd/C, 10%) and N, N’-dicyclohexylcarbodimide (DCC) were purchased from Kermel Chemical Reagent Co, Ltd (Tianjin, China). Heat-inactivated fetal bovine serum (FBS), Dulbecco’s modified Eagle’s medium (DMEM), 2, 4, 6-trinitrobenzenesulfonic acid (TNBS, 5%, w/v) and hexadecyltrimethylammonium bromide (HTAB) were purchased from Aladdin Bio-Chem Technology Co Ltd (Shanghai, China). Total superoxide dismutase (SOD), glutathione (GSH), malondialdehyde (MDA), glutathione peroxidase (GSH-Px) and myeloperoxidase (MPO) kits were purchased from Jiancheng Bioengineering Institute (Nanjing, China). The human epithelial colorectal adenocarcinoma cell line (Caco-2) was kindly provided by Stem Cell Bank, Chinese Academy of Sciences (Shanghai, China).

Male Kunming mice (20-25 g) and male Sprague-Dawley rats (220-250 g) were obtained from Laboratory Animal Center of Xi’an Jiaotong University, SCXK 2007-001 (Shaanxi, China). They were raised at a constant room temperature (22-25 ^º^C) under a 12 hr light-dark cycle, with free access to normal chow and water. Animals were allowed to acclimatize for one week before the start of experiments. All experimental protocols and animal care were in strict accordance with the Guidelines of the Laboratory Animal Center of Xi’an Jiaotong University and approved by the Institutional Animal Care and Use Committee of Xi’an Jiaotong University (No. XJTULAC2016-004).

***Synthesis of 5-ASA-ALA***

5-NSA (5 g, 27.3 mmol) was dissolved in anhydrous ethyl acetate (170 ml), DCC (7.2 g, 35 mmol) was dropwise added into the above solution with stirring at 0 ^°^C for 2 hr, and then alanine methyl ester (3.1 g, 30 mmol) was added into the mixture and stirred at room temperature for 10 hr. After completion of the reaction, the mixture was filtered and extracted with a saturated solution of sodium bicarbonate for three times. The combined extract was acidified with hydrochloric acid, extracted with ethyl acetate, and dried with anhydrous sodium sulfate, finally the organic solvent was removed by vacuum evaporation (13). The obtained precipitate was loaded on a silica gel column and eluted with chloroform/methanol (100:5), the eluate containing 5-nitrosalicyl-alanine was collected and the 5-nitrosalicyl-alanine methyl ester was obtained and concentrated by vacuum evaporation (4.4 g, yield: 60%).

5-Nitrosalicyl-alanine methyl ester (2.7 g, 10.0 mmol) and Pd/C (10%, 200 mg) were dissolved in methanol (30 mL), the solution was placed in a Parr pressure reactor and hydrogenated at 50 psi for 3 hr. After the reaction was completed, 5-aminosalicy-alanine methyl ester was obtained by filtration and dried by vacuum evaporation. Then the product was dissolved in sodium hydroxide solution (30 ml, 1M) and reacted for 2 hr under nitrogen protection. The pH value of the mixture was adjusted to pH 3-4, and finally, a crystallized white solid was obtained (5-ASA-ALA). 

***Characterization of 5-ASA-ALA***

The structure of 5-ASA-ALA was characterized by ^1^H NMR and FTIR. The ^1^H NMR analysis (in DMSO-d6) was conducted by a Varian 400 spectrometer (Varian, Palo Alto, CA, USA) at 400 MHz, the tetramethylsilane was used as an internal standard. The FTIR spectrum was recorded on an FTIR-8400s infrared spectrophotometer (Shimadzu, Kyoto, Japan), samples were uniformity mixed with the potassium bromide (1:100) and then used for analysis.

***Analytical method***

The concentration of 5-ASA-ALA and 5-ASA in experimental samples were detected by HPLC (Waters, Milford, MA, USA) using a BDS Hypersil C18 chromatographic column. The mobile phase was a mixture of phosphate buffer (pH=6.5) and methanol (85:15, v/v) containing 0.5% tetrabutylammonium chloride (14). The flow rate was 1.0 ml/min and the wavelength for a Waters2996 diode array detector (Waters, Milford, MA, USA) was 330 nm. The linearity of concentrations for 5-ASA and 5-ASA-ALA was obtained by linear regression, the absolute recovery of each compound was calculated by analyzing one sample at three concentration levels (0.4, 1.6 and 6.4 μg/ml) for five times, the precision of samples at different concentrations was evaluated by analyzing one sample five times within one day and five days, respectively, the relative standard deviation (RSD) was calculated accordingly.

***Stability study of 5-ASA-ALA***

This study was tested at three concentration levels of 5-ASA-ALA to evaluate its *in vitro* stability in tissue samples and validate the HPLC method for 5-ASA-ALA analysis under different conditions. Briefly, five rats were anesthetized and sacrificed, and their abdomen was cut open through the middle. Their stomach contents, small intestine contents and colon contents were collected and mixed with hydrochloric acid buffer (pH=1.2) and phosphate buffered saline (PBS) (pH=6.8 and 7.4), respectively, and homogenized to 20% (w/v). Then 1.0 ml of 5-ASA-ALA solution (dissolved in corresponding buffers) was mixed with the same volume of rat plasma, freshly prepared stomach contents, small intestine contents and colon contents, respectively, with the final concentration of 0.4, 1.6 and 6.4 μg/ml. Samples were divided into two groups, stored at room temperature for 10 hr or went through the freezing-thawing cycles twice. At the end of the storage and the freezing-thawing cycle, samples were centrifuged at 13000 g for 10 min and mixed with methanol (0.4 ml), then the mixture was vortexed and centrifuged again. Finally, the supernatant was collected, concentrated with nitrogen, and dissolved in methanol for HPLC analysis (15). Each concentration level was tested five times and the RSD was calculated respectively.

***Transport study of 5-ASA-ALA in Caco-2 cells***

Caco-2 cells were grown in DEME medium containing 10% FBS, 100 unit/ml peniciline, 100 µg/ml streptomycine, 2 mM glutamine, 1% sodium pyruvate and 1% non-essential amino acids at 37 ^°^C in a humidified atmosphere of 95% air and 5% CO_2_. Cells were seeded on polycarbonate membranes (Transwell, diameter 24 mm, pore size 0.4 μm, Corning Costar, NY, USA) at 1×10^5 ^cells/well in a 6-well plate (16). Propranolol was used as a positive control.

Eight days later, Caco-2 cells reached 100% confluence and then were cultured for 18 days more. The culture medium was discarded, and then the apical (AP) and basal (BL) chambers were washed for 3 times with PBS. The culture medium was then replaced with 1 ml of propranolol or 5-ASA-ALA solution (1 μmol/l) in the AP chamber. In the BL chamber, 2 ml of PBS was added instead of the culture medium. The plate was incubated at 37 ^°^C in an atmosphere of 95% air and 5% CO_2_ (17). After 0, 2, 4, 6, and 8 hr of incubation, the concentration of propranolol and 5-ASA-ALA in the BL chamber was measured by HPLC method, and the apparent permeability coefficient (P_app_) of drugs was calculated as follows: 

$P_{\mathrm{app}}\left(\frac{\mathrm{cm}}{s}\right)=\frac{\mathrm{dQ}}{\mathrm{dt}}\times \frac{1}{A\times C_{0}}$

where dQ/dt (μmol/s) is the transport rate, C_0_ (μmol/cm^3^) is the initial concentration in the donor chamber, and A (cm^2^) is the surface area of insert filter membrane. Experiments were performed in triplicate.

***Pharmacokinetic study of 5-ASA-ALA in rats***

Twelve male Sprague-Dawley rats were randomly divided in two groups (n=6), the 5-ASA-ALA group and 5-ASA group. Rats were orally administered with a certain amount of 5-ASA-ALA (equivalent to 50 mg/kg of 5-ASA) and 5-ASA. At 0.25, 0.5, 1, 2, 4, 6 and 8 hr, blood samples were collected from the jugular vein and then centrifuged at 13000 g, 4 ^°^C for 5 min to separate plasma. The plasma sample (0.1 ml) was mixed with methanol (0.4 ml), vortexed for 5 min and then centrifuged again. Finally, the supernatant was obtained, dried under nitrogen, dissolved in methanol and used for HPLC detection. 

***In vitro and in vivo incubation study of 5-ASA-ALA***

*In vitro incubation with contents of stomach and small intestine*

Rats (n=5) were anesthetized and sacrificed, their stomach contents and small intestine contents were collected and homogenized, and the homogenate was diluted to 20% (w/v) with different buffers (pH=1.2 hydrochloric acid buffer for stomach contents and pH=6.8 PBS for small intestine contents). One ml of the diluent was then mixed with the same volume of 5-ASA-ALA solution (equivalent to 100 μg/ml of 5-ASA, dissolved in various buffers) and incubated at 37 ^°^C. After 8 hr of incubation, the mixture was centrifuged, and the obtained supernatant was filtered. Finally, the concentration of 5-ASA released from 5-ASA-ALA in each sample was analyzed by HPLC.

*In vitro incubation with colon contents of rats*

Four g of colon contents of rats (n=6) were collected and mixed with 5-ASA-ALA solution (0.1 g/ml) in PBS (pH=7.4, 40 ml), the mixture was incubated under nitrogen atmosphere at 37 ^°^C. At the time point of 0, 1, 2, 3, 4, 5, 6, 7, 8, 9 and 10 hr, the mixture (0.5 ml) was sampled and replaced with an equivalent volume of PBS. Samples were then centrifuged and filtered, the concentration of 5-ASA was detected by HPLC method. The experiment was conducted at seven concentration levels of 5-ASA-ALA (27.5, 50.0, 72.5, 105.0, 150.0, 200.0 and 305.0 μg/ml). The hydrolysis curve of each concentration was drawn, the linear regression was made to obtain regression equations, and the slope of each curve was regarded as the hydrolysis rate of 5-ASA-ALA.

*In vivo incubation in the colon of rats*

Rats were randomly divided into two groups (n=6), the clindamycin group and the control group. Clindamycin was used as an inhibitor of the colonic microflora. Rats were administered with a certain dose of clindamycin (25 mg/kg of body weight) and an equivalent volume of normal saline by gavage, twice daily for three days, respectively. After that, rats were anesthetized by diethyl ether, their abdomen was cut open, and then 5-ASA-ALA solution (2 mg, dissolved in 1 ml of PBS, pH=7.4) was injected into their colon, finally, their colon was tied at both ends and their abdomen was sutured immediately (18, 19). Eight hr later, animals were sacrificed, the colon contents of rats were collected and homogenized in PBS (pH=7.4, 40 ml). The homogenate was then centrifuged, and the supernatant was obtained and mixed with four times the volume of methanol, finally, samples were centrifuged, filtered and analyzed by HPLC. The percent of hydrolysis of 5-ASA-ALA was calculated as follows:

$\mathrm{percent of hydrolysis}\left(\%\right)=(1-\frac{W_{f}}{W_{t}})\times 100$

where W_f_ is the free amount of 5-ASA in the colon contents and W_i_ is the initial amount of 5-ASA-ALA that administered to rats.

***Therapeutic effects of 5-ASA-ALA on TNBS-induced colitis***

*Experiment protocols and colitis induction*

Colitis induction was performed based on the previous study (20). After a 12 hr of fast, mice were anesthetized with pentobarbital sodium (40 mg/kg of body weight). TNBS solution (0.1 ml dissolved in 50% ethanol, 2.5%) was injected into the colon of mice (3.5-4 cm from the anus), and then mice were kept head downward for 30 sec to prevent drug leakage. Twenty-four hr after the injection of TNBS, mice were administrated once daily for five continuous days with different drugs. Briefly, mice in treatment groups were administrated with a given dose of drugs by gavage (0.1 ml, equivalent to 100 mg/kg of 5-ASA) (21). Besides, mice in the control group and the positive group received a same volume of normal saline, and a physical mixture of 5-ASA and alanine, respectively. Forty-eight mice were randomly divided into four groups (n=12): 1) control (intragastric-gavage (ig), normal saline, no colitis induced), 2) TNBS (ig, normal saline), 3) positive (ig, 100 mg/kg 5-ASA+56 mg/kg alanine, dissolved in normal saline), 4) 5-ASA-ALA (ig, 156 mg/kg, dissolved in normal saline). 

*Survival rate and body weight of mice*

From day 0 of colitis induction, the body weight of each mouse and the death of each group were recorded daily. Finally, the degree of TNBS-induced colitis in mice of each group was evaluated based on the curves of weight changes and the final survival rate of mice for each group.

*Disease activity index (DAI) score of mice*

The disease activity index (DAI) of mice was scored and recorded daily from day 1 according to the scoring criteria (Table 1). DAI is a sum of scores for three indexes and it is ranged from 0 to 4 depending on the severity. The degree of fecal occult blood was evaluated by the ο-tolidine method (22), and scored based on the criteria reported by Kohn and kelly (23).

*CDS and colon index of mice*

All mice were sacrificed on the 5^th^ day of the experiment, their colonic tissues were excised, cut longitudinally, washed carefully with normal saline, and observed with a magnifying glass. The colonic damage score (CDS) was evaluated according to following scoring criteria: 0, no ulcer and colitis; 1, local hyperemia without ulcer; 2, ulcers without local hyperemia; 3, only one ulcerative and inflammatory lesion; 4, more than one ulcerative and inflammatory lesions; 5, more than 2 cm of continuous ulcer areas (24).

The entire colon samples were dried with filter papers, the length and weight of the samples were measured and recorded, respectively, in order to calculate the colon index of mice as the following formula:

Colon index (g/dm) = weight of colon/length of colon

*MPO activity in colonic tissues*

Colonic tissue samples were cleaned, dried and then suspended in HTAB solution (0.5%, pH=7.4), the final concentration of the tissue samples was 50 g/l. Samples were homogenized at 4 ^°^C, sonicated in an ice bath for 10 sec, underwent the freeze-thawing cycle twice, and then sonicated again. The homogenate was centrifuged at 4 ^°^C, 13000 *g* for 15 min, and then MPO was extracted from the resulting supernatant (25). Finally, MPO activity in colonic tissues was detected by an assay kit according to the provider’s instructions.

*Levels of GSH, MDA, GSH-Px and SOD in colonic tissues*

Clean and dried colonic tissues were homogenized in ice-cold normal saline, the resulting homogenate (10%, w/v) was centrifuged at 4 ^°^C, 13000 *g* for 15 min. The obtained supernatant was diluted with normal saline to 1% (w/v), and the protein levels in the supernatant were measured by a BCA protein assay kit. The levels of GSH and MDA, and the activity of GSH-Px and SOD in the colonic tissues were measured by assay kits according to the provider’s instructions. 

***Statistical analysis***

Statistical analyses were performed with SPSS Version 19.0 (IBM Corporation, Armonk, NY, USA). All data with normal distribution were presented as mean±standard deviation (SD). One-way analysis of variance followed by the Fisher’s least significant difference test was used. Statistically significant differences were accepted as *P*<0.05.

## Results

***Preparation and characterization of 5-ASA-ALA***

**Figure 1:**
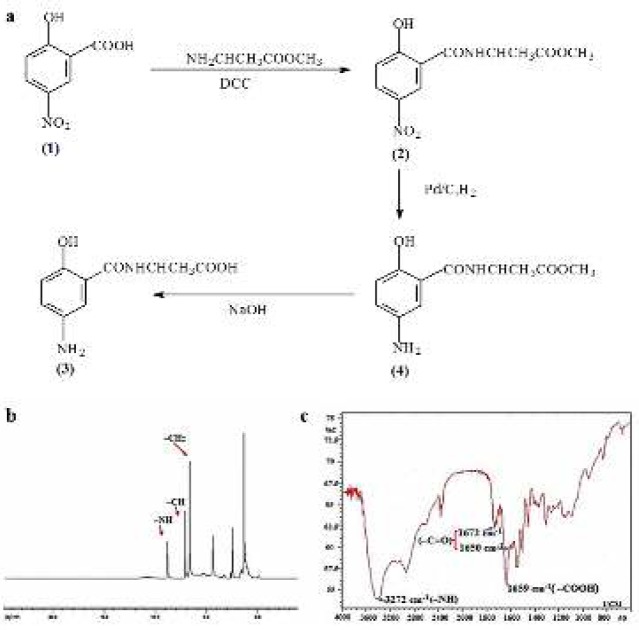
Synthesis and characterization of 5-ASA-ALA. (a) The synthetic route of 5-ASA-ALA. (b) The 1H NMR spectrum of 5-ASA-ALA. (c) The FTIR spectrum of 5-ASA-ALA. 5-ASA-ALA, 5-aminosalicylic acid-alanine

The synthetic procedure of 5-ASA-ALA is shown in Figure 1a. The amino group of alanine methyl ester was reacted with the carboxyl group of 5-NSA **(1)** and catalyzed by DCC to obtain 5-NSA-ALA methyl ester **(2)**. The nitro group of **(2)** was reduced under the action of hydrogen and Pd/C, and then the ester bond of **(3)** was hydrolyzed by sodium hydroxide and the final product 5-ASA-ALA **(4)** was obtained. 5-ASA-ALA was characterized by ^1^H NMR and FTIR (Figure 1b and 1c). Data indicated that 5-ASA-ALA was successfully synthesized. Yield: 2.2 g, 80%. mp: 190-194 ^°^C. IR (KBr), ν_max_ (cm^-1^): 1650 (C=O), 1672 (C=O), 1659 (-COOH), 3272 (-NH-). ^1^H NMR (DMSO-d6): δ 6.69 (s, 2H, ArH), 7.13 (s, 1H, ArH), 8.86 (s, 1H, -NH-).

***HPLC analytical method***

After analyzing the peak area of each sample and its concentration (0.5-20.0 μg/ml) by regression statistics, linear regression equations of 5-ASA-ALA and 5-ASA in tissue samples for HPLC analysis were established and correlations were examined as well. As shown in Table 2, absolute recoveries of 5-ASA-ALA and 5-ASA at three concentration levels (0.4, 1.6 and 6.4 μg/ml) were >90%, and there was no signiﬁcant difference in recoveries between the three concentrations. In precision assay, RSD of both inter- and intra-day for all samples were <7% (Table 3). Data demonstrated that the HPLC analytical methods were accurate, reproducible and reliable.

***Stability study of 5-ASA-ALA***

The *in vitro* stability of 5-ASA-ALA in different tissue samples was evaluated at three concentration levels under two conditions, stored at the room temperature and suffered the freezing and thawing. After storage under different conditions, the concentrations of 5-ASA-ALA were analyzed for calculating the RSD of each sample. As shown in Table 4, the RSDs of 5-ASA-ALA in all tissue samples (at 0.4, 1.6 and 6.4 μg/ml) were <4%, which suggested the remarkable stability of 5-ASA-ALA in rat plasma and in the imitative environment of gastrointestinal tract.

***Transport of 5-ASA-ALA across Caco-2 monolayers***

The transepithelial Caco-2 model was used to assess the transport characteristic of 5-ASA-ALA and further evaluate its potential for passive extraction and diffusion from the small intestine (26). Propranolol was used as the positive control because of its good absorption in Caco-2 cell monolayer with a P_app_ of 2.75×10^-5^ cm/sec. In this study, the detected P_app_ of propranolol was (2.48±0.77)×10^-5^ cm/sec at 100 μmol/l, which suggested that the permeability of Caco-2 model in this study met the experimental condition. In the apical to basal direction, the P_app_ for 5-ASA-ALA was (2.63±0.86)× 10^-6^ cm/sec and (1.73±0.12)×10^-6^ cm/sec at 200 and 400 μmol/l, respectively. The absorption of 5-ASA-ALA was significantly less than the propranolol in the Caco-2 monolayer.

***The pharmacokinetic study of 5-ASA-ALA***

**Figure 2:**
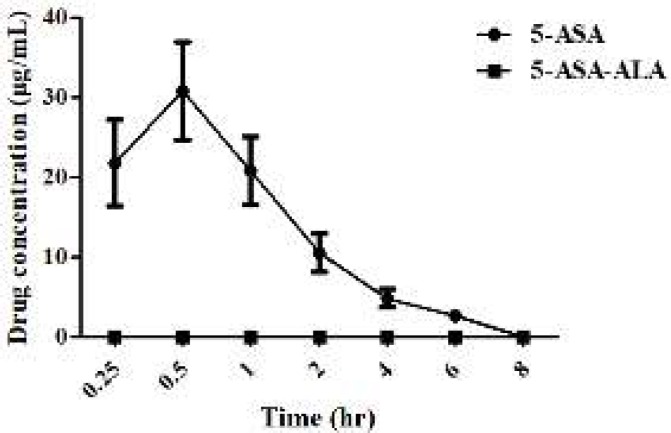
Plasma concentration curves of 5-ASA and 5-ASA-ALA in rats. 5-ASA-ALA, 5-aminosalicylic acid-alanine; 5-ASA, 5-aminosalicylic acid. Data are means±SD, n=6

The pharmacokinetic parameters of 5-ASA-ALA and 5-ASA are shown in Figure 2. The T_max_ of 5-ASA was 0.5 hr after oral administration, indicating its fast absorption and high bioavailability. During the experimental period (0-8 hr), the plasma concentration of 5-ASA-ALA was not detected, which was probably because its concentration was far lower than the detection limit of this analytical method. This result indicated that the drug absorption into the blood was significantly reduced by 5-ASA-ALA, thus further minimizing the systemic absorption and greatly ameliorating the side effects of drugs.

***In vitro and in vivo incubation study of 5-ASA-ALA***

**Figure 3:**
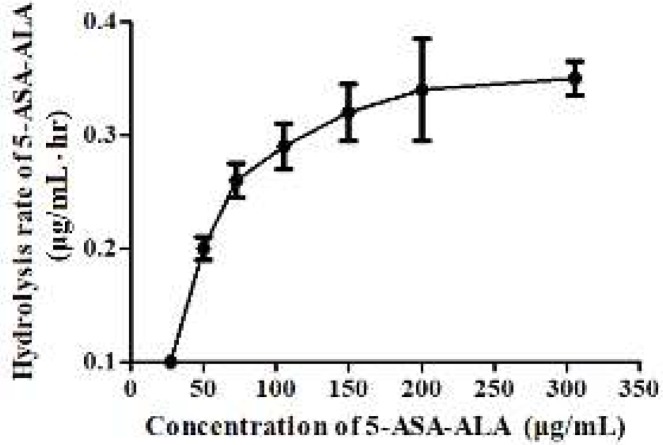
Hydrolysis rate of 5-ASA-ALA during incubation with the colon contents of rats. 5-ASA-ALA, 5-aminosalicylic acid-alanine. Data are means±SD, n=6

**Figure 4:**
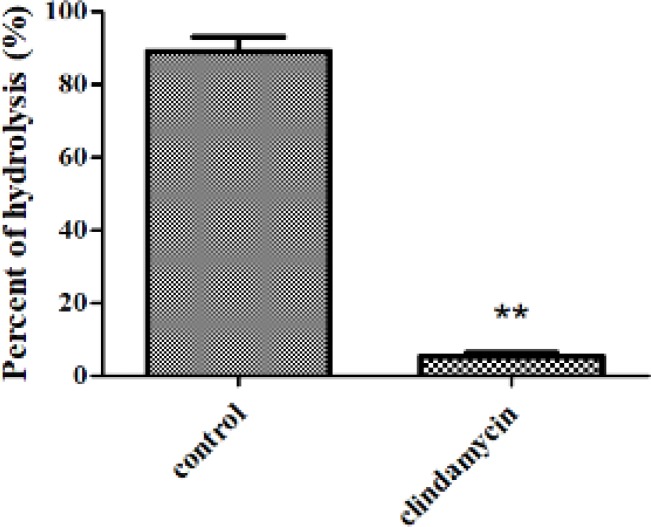
Percent of hydrolysis of 5-ASA-ALA in clindamycin group and control group after in vivo incubation in rats for 8 hr. 5-ASA-ALA, 5-aminosalicylic acid-alanine. Data are means±SD, n=6, **P<0.01

5-ASA-ALA was incubated with contents of stomach, small intestine and colon to investigate the hydrolysis behavior of 5-ASA from 5-ASA-ALA in the gastrointestinal tract of rats. As a result, there was no detectable 5-ASA in stomach contents and small intestine contents of rats during the experimental period, which indicated its remarkable stability in the upper gastrointestinal tract environment. However, 5-ASA was gradually released from 5-ASA-ALA when incubated with colon contents of rats, and its hydrolysis rate against different substrate concentration was calculated. As shown in Figure 3, the hydrolysis rate was sharply increased and in direct proportion to the concentration of 5-ASA-ALA at lower concentration, which followed the first-order reaction. With further increase of the substrate concentration, its hydrolysis rate increased slowly and then reached a saturated value. When the concentration of 5-ASA-ALA was above 200 µg/ml, the hydrolysis rate did not increase anymore and presented as a zero-order reaction because the enzyme secreted by colonic microflora was saturated at this substrate concentration. As shown in Figure 4, 89.14% of 5-ASA-ALA was hydrolyzed in the control group after incubation in rats for 8 hr. However, in the clindamycin group, the hydrolysis behavior of 5-ASA-ALA was significantly decreased (*P*<0.01) under the inhibitory effect of clindamycin with 5.26% of hydrolysis. These results suggested that the hydrolysis of 5-ASA-ALA was strongly associated with the activity of colonic microflora.

***Effect of 5-ASA-ALA on survival rate and body weight of mice***

**Figure 5:**
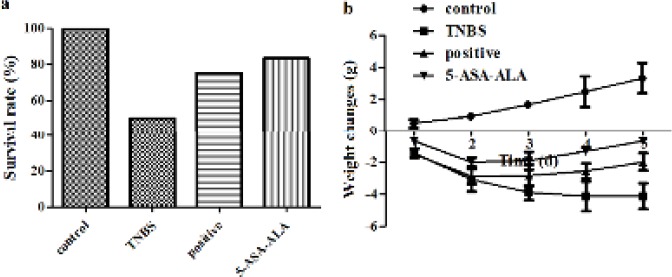
Effects of 5-ASA-ALA on survival rate (a) and body weight (b) of mice. The weight loss of mice in each group was recorded daily for 5 days continuouslly, the survival rate in each group was calculated at the end of the experiment. A physical mixture of 5-ASA and alanine was used as the positive control. 5-ASA-ALA, 5-aminosalicylic acid-alanine. Data are means±SD, n=12

The effect of 5-ASA-ALA on experimental colitis was firstly evaluated by the body weight and survival rate of mice. As shown in Figure 5a, expect for the control group, deaths of mice were observed in all groups during the experiment, especially, the TNBS group was the most severe (50.0%). But there was no significant difference between the positive control and the 5-ASA-ALA group in survival rate, which could be because of the small number of animals (n=12). Mice in the control group presented a steady increasing trend of body weight, while the body weight of the TNBS group was continuously reduced (Figure 5b). The results indicated that TNBS caused severe colitis in mice, the colitis model of mice is successfully established. On the contrary, the 5-ASA-ALA group and the positive group showed a slight weight loss in the first two days, while the situation was gradually alleviated from the third day, suggested that 5-ASA-ALA had positive effect on the treatment of UC.

***Effect of 5-ASA-ALA on DAI score***

**Figure 6:**
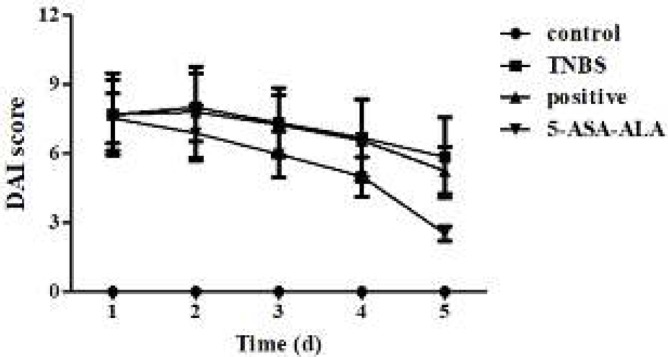
Effects of 5-ASA-ALA on DAI of mice. After the induction of colitis, mice in each group were administered with drugs equivalent to 100 mg/kg of 5-ASA. The DAI of mice was recorded and scored daily. A physical mixture of 5-ASA and alanine was used as the positive control. 5-ASA-ALA, 5-aminosalicylic acid-alanine; DAI, disease activity index. Data are means±SD, n=12

After the induction of colitis with TNBS, mice exhibited classic symptoms of UC, such as huddle, hypokinesia, anorexia, diarrhea, bloody stools, and various levels of weight loss. Compared with the control group, this ultimately led to a steep increase of DAI on the first day (Figure 6). Drugs were given once daily for five continuous days to mice in order to predict the therapeutic effect of 5-ASA-ALA. As a result, DAI of mice in treatment groups was significantly reduced from the third day, especially, 5-ASA-ALA was more effective than the positive control.

***Effect of 5-ASA-ALA on CDS and colon index of mice***

**Figure 7:**
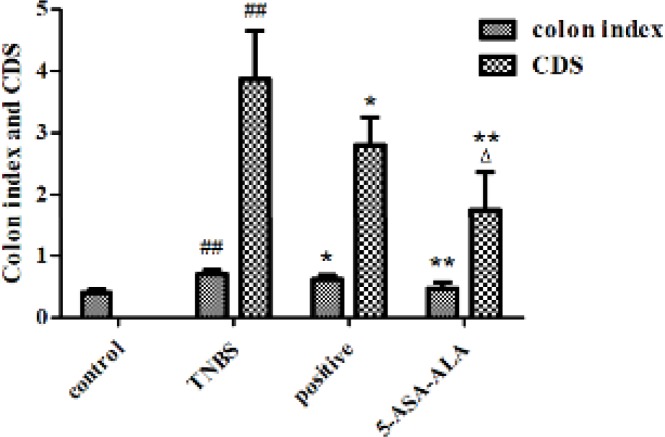
Effects of 5-ASA-ALA on colon index and CDS of mice. (a) At the end of the experiment, all mice were sacrificed and their entire colons were excised and measured to calculate the colon index. (b) The CDS of mice in each group was scored according to the macroscopical changes of the colon. CDS, colonic damage score. ##: *P<*0.01 vs control group; *: *P<*0.05, **: *P<*0.01 vs TNBS group; ∆: *P<*0.05 vs positive control group. Data are shown as mean±SD, n=12

After dissection, the mucosa damage of colon tissues in mice was scored and evaluated. Observation showed that, the entire colon of TNBS-treated mice was involved by inflammatory lesions, the main manifestations were deformation, edema, necrosis and transmural injury of the colon. As shown in Figure 7, CDSs of treatment groups were significantly lower than the TNBS group, while TNBS group showed obviously higher CDS when compared with the control group (*P*<0.01). Besides, 5-ASA-ALA presented a significantly greater effect than the positive control (*P*<0.05).

TNBS-induced experimental colitis may lead to compensatory hypertrophy in colonic tissues, which could result in prominent colonic wall thickening and shortening of the colon length, ultimately, it might cause an increase of colon index. After treatment, the colon index of mice was significantly reduced when compared with the TNBS group (Figure 7, *P*<0.01). The TNBS group showed a significantly higher colon index than the control group (*P*<0.01), while the 5-ASA-ALA group presented a similar result to the control group.

***Effect of 5-ASA-ALA on MPO activity in colonic tissues***

**Figure 8:**
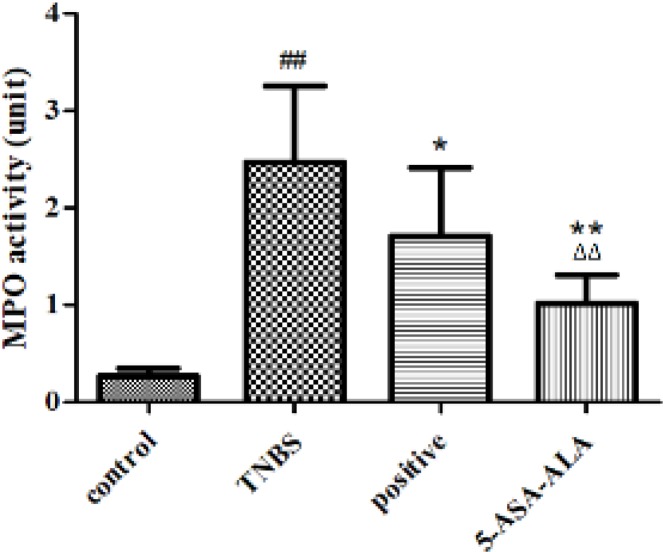
Effects of 5-ASA-ALA on MPO activity of mice. After treatment, the MPO activity of mice in colonic tissues was measured to evaluate the potential therapeutic effects of 5-ASA-ALA. MPO, myeloperoxidase. ##: *P<*0.01 vs control group; *: *P<*0.05, **: *P<*0.01 vs TNBS group; ∆∆: *P<*0.01 vs positive control group. Data are mean±SD, n=12

MPO is an important lysosomal protein and peroxidase enzyme that most abundantly expressed and stored in azurophilic granules of the neutrophil, during degranulation, it is released into the extracellular space. Therefore, MPO is used for quantitative determination of inflammation because of the association between the elevated MPO level and the severity of UC. As shown in Figure 8, when compared with the TNBS group, 5-ASA-ALA significantly reduced the MPO activity in colonic tissues (*P*<0.01), and it was obviously more effective than the positive control (*P*<0.01).

***Levels of GSH, MDA, GSH-Px and SOD in colonic tissues***

**Figure 9:**
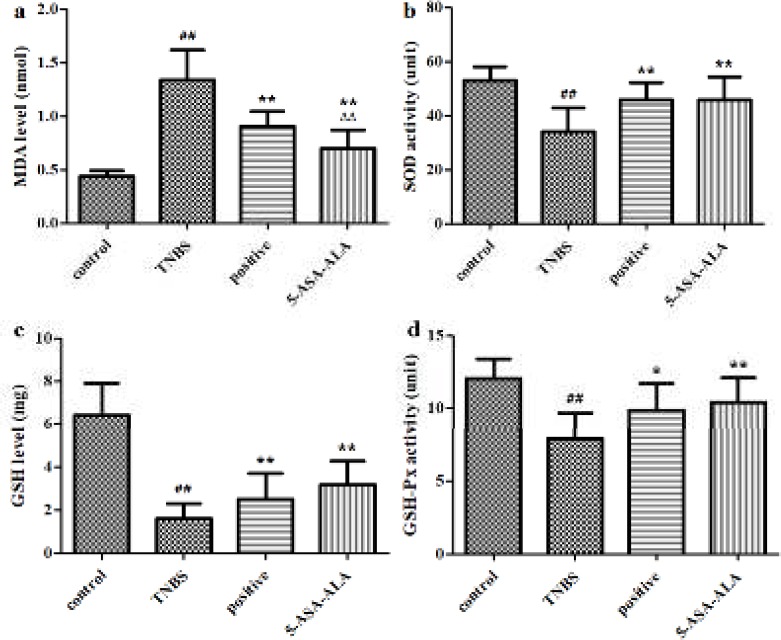
Effects of 5-ASA-ALA on levels of MDA, SOD, GSH and GSH-Px. After treatment, the MDA level (a), SOD activity (b), GSH level (c) and GSH-Px activity (d) of mice in colonic tissues were measured to evaluate the potential therapeutic effects of 5-ASA-ALA. SOD, superoxide dismutase; MDA, malondialdehyde; GSH, glutathione; GSH-Px, glutathione peroxidase. ##: *P<*0.01 vs control group; *: *P<*0.05, **: *P<*0.01 vs TNBS group; ∆∆: *P<*0.01 vs positive control group. Data are mean±SD, n=12

As shown in Figure 9, the TNBS-induced colitis significantly increased the MDA level (*P*<0.01), significantly decreased the GSH level, GSH-Px activity and SOD activity (*P*<0.01) as compared with the control group. After five days of treatment with 5-ASA-ALA, mice showed a significantly lower MDA level (*P*<0.01), a significantly higher GSH level and higher activities of GSH-Px and SOD (*P*<0.01) in colonic tissues than mice in the TNBS group. Besides, 5-ASA-ALA presented a similar therapeutic effect to that of the positive control, and it showed significantly greater effect on reducing the MDA level (*P*<0.01) in the colon than the positive control.

**Table 1:** The evaluation criteria of disease activity index (DAI) of mice

Weight loss	Stool consistency	Fecal occult blood	Score
none	well-formed pellets	normal	0
1%-5%	loose stools	occult blood +	1
5%-10%		occult blood ++	2
10%-15%	diarrhea	occult blood +++	3
> 15%		visible gross bleeding	4

**Table 2:** Linear equations of 5-ASA-ALA and 5-ASA in different tissue samples for HPLC analysis

Samples		Equations	R^2^	LOQ (μg/mL)
5-ASA-ALA	plasma	y=4.9727x-2.623	0.9990	0.5
	stomach contents	y=5.0283x-2.702	0.9992	0.5
	small intestine contents	y=5.2218x-2.753	0.9996	0.5
	colon contents	y=4.8049x-2.446	0.9998	0.5
5-ASA	plasma	y=7.1343x-1.479	0.9993	0.5
	stomach contents	y=8.7111x-0.081	0.9995	0.5
	small intestine contents	y=7.9948x-0.693	0.9988	0.5
	colon contents	y=6.9505x-0.584	0.9986	0.5

5-ASA-ALA, 5-aminosalicylic acid-alanine; 5-ASA, 5-aminosalicylic acid

**Table 3:** Recovery and precision for 5-ASA-ALA and 5-ASA detection in different tissue samples by HPLC (means±SD, n=3)

Samples		Concentration (μg/mL)	Recovery (%)	Intra-day RSD (%)	Inter-day RSD (%)
5-ASA-ALA	plasma	0.4	94.4±3.4	3.6	4.8
		1.6	97.5±4.1	2.6	3.3
		6.4	103.1±4.8	2.1	3.5
	stomach contents	0.4	108.0±2.3	3.8	2.4
		1.6	92.5±3.6	4.6	4.5
		6.4	95.0±4.5	4.9	6.6
	small intestine contents	0.4	105.0±3.5	2.2	4.0
		1.6	96.0±4.4	3.2	5.6
		6.4	96.3±4.5	4.6	3.3
	colon contents	0.4	104.0±2.5	4.1	4.3
		1.6	97.5±3.8	2.8	2.9
		6.4	95.0±3.5	2.7	3.8
5-ASA	plasma	0.4	107.5±5.1	4.6	4.8
		1.6	99.4±3.4	3.2	3.6
		6.4	101.6±4.8	2.1	3.3
	stomach contents	0.4	105.0±2.3	3.1	2.4
		1.6	101.9±2.6	2.6	2.6
		6.4	96.9±1.5	1.9	1.5
	small intestine contents	0.4	107.5±2.4	3.2	3.3
		1.6	96.6±2.5	2.2	2.0
		6.4	99.4±3.5	2.7	1.8
	colon contents	0.4	105.0±3.5	2.8	3.9
		1.6	98.1±2.8	2.7	2.8
		6.4	101.6±2.5	2.1	1.8

5-ASA-ALA, 5-aminosalicylic acid-alanine; 5-ASA, 5-aminosalicylic acid; HPLC, high performance liquid chromatography; RSD, relative standard deviation

**Table 4:** Stability study of 5-ASA-ALA in different tissue samples under normal and freezing-thawing conditions (n=5)

Samples	Concentration (μg/mL)	RSD (%)	
		storage at room temperature for 10 hr	freezing and thawing twice
plasma	0.4	2.2	2.5
	1.6	1.9	2.2
	6.4	1.1	1.5
stomach contents	0.4	1.7	1.9
	1.6	1.8	1.5
	6.4	1.3	1.5
small intestine contents	0.4	3.3	2.6
	1.6	3.5	2.0
	6.4	2.0	1.8
colon contents	0.4	2.7	2.0
	1.6	2.2	1.7
	6.4	1.6	1.2

5-ASA-ALA, 5-aminosalicylic acid-alanine; 5-ASA, 5-aminosalicylic acid; RSD, relative standard deviation

## Discussion

5-ASA is the most commonly used drug for the treatment of UC. However, after oral administration, 5-ASA undergoes quick and almost complete absorption from the upper gastrointestinal tract, and the drug concentration in the colon is greatly reduced (27). In this study, a prodrug of 5-ASA was prepared by using the 5-NSA as a starting material, by this means, the synthesis process was simpler and the yield was higher than using 5-ASA as the starting material (28). Besides, 5-ASA-ALA was formed from an amidation reaction and possessed a remarkably stable structure, it had the potential to be hydrolyzed in the colon by colonic microflora and hence released 5-ASA to reduce inflammation at the ulcer lesion. This could be because 5-ASA-ALA comprises secondary amine groups, the rat colon microflora probably produces relevant enzyme systems (specific secondary amine deaminases) in the colon, which are effective in hydrolyzing amine bonds. Our study suggested that 5-ASA-ALA may bypass the upper gastrointestinal tract, and remained stable in the blood, but it would be gradually hydrolyzed in the colon. This approach may, therefore, be used as a particular system for targeting delivery of 5-ASA in UC treatment.

Specifically, the pharmacokinetic study showed that the drug absorbed into the blood was significantly reduced by 5-ASA-ALA. The most common side effects of 5-ASA include diarrhea, pericarditis, pancreatitis, allergic reaction and renal toxicity, which are strongly dose-related, therefore, the side effects caused by systemic absorption could be greatly ameliorated. The transepithelial transport study indicated that the absorption of 5-ASA-ALA from the small intestine would be minimum and the prodrug would remain stable in the upper gastrointestinal tract, which also indicating that 5-ASA-ALA could effectively decrease the drug absorption of 5-ASA. Furthermore, the *in vitro* and *in vivo* incubation study were conducted to evaluate the colon-targeting effect of 5-ASA-ALA. In the *in vitro* study, 5-ASA-ALA remained intact in the environment of upper gastrointestinal tract but gradually hydrolyzed in colon contents, besides, its hydrolysis rate presented as a typical enzymatic reaction, which showed the importance of an appropriate concentration of enzyme inhibitor in the incubation media. The *in vivo* incubation study indicated that the hydrolysis of 5-ASA-ALA was strongly associated with the colonic microflora, this hydrolysis behavior would lead to a longer retention time in the gastrointestinal tract after oral administration, because there are abundant microorganisms being activated in the lower gastrointestinal tract, especially in the colon where the prodrug could be hydrolyzed specifically. These results suggested that 5-ASA-ALA has great potential as an oral colon-targeting prodrug of 5-ASA.

The therapeutic effect of 5-ASA-ALA on TNBS-induced colitis in mice was estimated by a series of indicators, which could effectively reflect the severity of inflammation. The health status of mouse models and the onset time of drugs were real-time monitored by the weight trends and DAI score of mice. After treatment, the levels of inflammation were directly reflected by the survival rate of mice, the CDS and the colon index of colonic tissues. The severity of colitis in mice was quantitatively reflected by the levels of MDA and GSH, and the activities of MPO, GSH-Px and SOD in colonic tissues, besides, these indicators could also help predict the pathogenesis of experimental colitis and the action mechanism of drugs. Observations showed that, deaths of mice occurred in all groups after the induction of colitis, especially on the third day. Results of dissection suggested that the primary cause of death was ileus resulted from acute colitis, the enterobrosis was seen in some dead mice. Some of the dead mice suffered from intestinal perforation caused by inflammation, in severe cases, mesenteric adhesion was very serious and could not be dissected. During the experiment, the TNBS-treated mice showed different degrees of weight loss, hypokinesia, anorexia, diarrhea and bloody stools. After five days of drug therapy, the above symptoms were gradually relieved in surviving mice, the disease related indicators were almost close to the control group.

The DAI score of mice in test groups continued to be increased in the first two days after the induction of colitis, and it was started to reduce to some different extent from the third day. After administration with drugs, the DAI of mice was significantly reduced from the third day, particularly, 5-ASA-ALA was the most effective. MPO is a functional and active marker of neutrophils, and its level and activity changes represent the status of neutrophilic polymorphonuclear leukocytes. The detection of MPO activity in intestinal tissues is a simple biochemical assay according to report by Krawisz *et al.*, MPO could serve as a useful marker and a sensitive predictor for acute inflammation in colonic tissues from animals with colitis (29). Elevated MPO levels may reflect the neutrophilic leukocytosis and the density of neutrophil infiltration. Declined MPO activity could be regarded as the quantification of anti-inflammatory effect for given drugs. In comparison with the TNBS group, 5-ASA-ALA significantly reduced the MPO activity in colon homogenate of colitis mice and produced a better result than the positive control.

SOD is an important antioxidant enzyme that widely distributed in nearly all living cells. SOD has special physiological activity and is the primary substance for scavenging free radicals, so that SOD could defense and block the damage caused by toxic superoxide radical to cells and repair damaged cells in time (30). In this study, the SOD activity in colonic tissues is a direct reflection of damage degree and inflammation development. MDA is one of the most important products of membrane lipid peroxidation, it can cause cross-linking polymerization of biomacromolecule and form covalent protein adducts, besides, it has cytotoxicity and could aggravate membrane damage. Therefore, the production of MDA is a common indicator of oxidative stress level in organisms (31). The MDA level in tissues could indirectly reflect the degree of lipid peroxidation and cellular damage. GSH is a tripeptide that exists in almost every cell of the body. GSH helps maintain the normal function of immune system and has antioxidant and integrated detoxification effects. Its main physiological function is to scavenge free radicals from the body, as a crucial antioxidant, GSH protects sulfhydryl groups in proteins and enzymes (32). In this study, the GSH level in tissues is regarded as an indicator of antioxidant ability and a direct reflection of the inflammatory damage degree in the colon. GSH-Px is an essential peroxidase widely existed in the body. It can reduce toxic peroxides into nontoxic hydroxyl compounds, while promoting the decomposition of hydrogen peroxide, thereby protecting the structure and function of cell membranes from interference and damage by peroxides (33).

Therefore, the ameliorative effect of 5-ASA-ALA on colitis in TNBS-induced mice was estimated through measuring the above indicators in colonic tissues, including the decrease in the level of MDA and the increase in activities of GSH-Px, SOD and GSH. Results suggested that, 5-ASA-ALA had obvious therapeutic effect on the experimental colitis compared to the TNBS group, and it was significantly more effective than the physical mixture. Moreover, data indicated that 5-ASA-ALA exerts its anti-inflammatory efficacy probably through anti-oxidative stress, while further study is needed to identify its action mechanism more profoundly.

## Conclusion

5-ASA-ALA was designed and synthesized as a prodrug of 5-ASA by a simple synthetic route with high yield, its structure was characterized by FTIR and ^1^H NMR. The stability study was conducted under normal and the freezing-thawing conditions, respectively, the result showed that 5-ASA-ALA was remarkably stable in rat plasma and the imitative environment of upper gastrointestinal tract. The transport study showed that few amount of 5-ASA-ALA was absorbed in the Caco-2 cell, therefore, its absorption from the small intestine was minimum. The *in vitro* and *in vivo *incubation study were performed to systemically estimate the colon-targeting property of the prodrug. Data showed that 5-ASA-ALA was stable in the upper gastrointestinal, but gradually hydrolyzed in the colon of rats, and its hydrolysis behavior was strongly associated with colonic microflora. The ameliorative effect of 5-ASA-ALA in TNBS-induced colitis mice was evaluated by a set of indicators. After oral administration to mice, 5-ASA-ALA showed satisfactory therapeutic effect and it was significantly more effective than the physical mixture of 5-ASA and alanine. All results indicated that 5-ASA-ALA could be as an oral colon-targeting prodrug of 5-ASA and may have potential application in the treatment of UC.
